# Dynamic Compressive Behavior of Graded Auxetic Lattice Metamaterials: A Combined Theoretical and Numerical Study

**DOI:** 10.3390/ma18225187

**Published:** 2025-11-14

**Authors:** Zeyao Chen, Jinjie Liu, Xinhao Li, Yixin Zhou, Zhihao Ou

**Affiliations:** School of Electro-Mechanical Engineering, Guangdong University of Technology, Guangzhou 510006, China; liujinjie@mails.gdut.edu.cn (J.L.); lixinhao@mails.gdut.edu.cn (X.L.); 2112101039@mail2.gdut.edu.cn (Y.Z.); 1112101028@mail2.gdut.edu.cn (Z.O.)

**Keywords:** auxetic lattice, metamaterials, gradient, dynamic compression, negative poisson’s ratio

## Abstract

Auxetic metamaterials, characterized by negative Poisson’s ratio, have garnered significant interest due to their exceptional impact resistance. This study presents a type of auxetic metamaterial organized in re-entrant arrowhead lattices. The uniaxial impact behavior of a uniform auxetic lattice was first investigated through experiment and finite element simulation, which showed good agreement. Subsequently, two graded auxetic lattices with density-gradient profiles were proposed by varying the radius of the bars in the basic auxetic lattice. Numerical simulations demonstrate that, across various compression velocities, both graded architectures achieve higher plateau stresses and enhanced energy absorption compared to their uniform counterpart. Notably, the graded lattice with lower density at the impact end exhibited a reduced initial peak stress. An analytical framework was also established to characterize the compressive behavior of these auxetic lattices. Theoretical analyses elucidate the underlying mechanisms of impact energy dissipation and provide a solid basis for predicting dynamic compressive performance. Furthermore, a gradient-parametric study revealed that the stress–strain response is significantly influenced by both the density gradient and impact velocity, further demonstrating a high consistency between the theoretical predictions and the simulation results. This research is desirable to provide insights for designing graded auxetic metamaterials with tailored impact properties.

## 1. Introduction

Inspired by natural cellular materials, artificial lightweight cellular materials, including lattices, foams, and sandwiches, have attracted significant attention because of their superior properties not only in terms of high specific stiffness and strength but also in terms of energy absorption capacity, heat transfer, vibration dissipation, acoustic properties, and multifunctional performance [1,2]. As a type of cellular material or structure, periodic lattices are constructed using regular unit cells consisting of rods or plates, showing superior performance compared to other random cellular materials [3,4]. Owing to the development of advanced manufacturing techniques such as three-dimensional (3D) printing, complicated lattice structures can be fabricated and systematically investigated [5]. The static mechanical properties of various periodic lattices have been investigated intensively [6,7,8,9]. Moreover, the dynamic mechanical performance of lattices has been studied using experiments and simulations, showing advantages in impact energy dissipation [10,11,12].

Auxetic materials contract transversely in compression but expand in tension [13]; this is called the negative Poisson’s ratio (NPR) effect [14]. Although auxetic materials with NPR were initially proposed in the form of foams [13], great attention has been paid to auxetic lattices [15,16,17], because of the high degree of designability of their mechanical properties. Benefiting from the rapid development of advanced additive manufacturing technologies, it is possible to fabricate sophisticated auxetic lattice structures [18,19,20,21,22]. The NPR property can be utilized to improve mechanical performance, especially the energy absorption capability [23]. Auxetic materials exhibit high energy absorption dissipation and impact resistance, making them superior in protective engineering [24,25]. Understanding the stress–strain relationships of auxetic cellular materials under quasi-static/dynamic compression can effectively guide the crashworthiness design of auxetic materials [26]. Chen et al. [27] proposed a 3D auxetic lattice with enhanced stiffness and investigated its quasi-static compressive properties through experiments and simulations. Yang et al. [28] reported an integrated theoretical, computational, and experimental investigation on the compression and impact of typical auxetic materials. Zhao et al. [29] deduced an empirical formula for the critical impact velocity and proposed a theoretical model to predict the dynamic crushing strength for a double-arrowed auxetic structure, which was validated by numerical results. Li et al. [30] introduced a re-entrant auxetic honeycomb as reinforcement for soft materials as the matrix to create a class of composites and found 3D printed auxetics composites can achieve a significant enhancement in their indentation stiffness and impact resistance compared with non-auxetic composites. Luo et al. [31] proposed a novel auxetic composite by filling re-entrant honeycombs with foam and investigated the enhanced effect of the auxetic structure on energy absorption under uniaxial crushing. In addition to the re-entrant configuration, auxetic chiral lattice composites have been found to exhibit enhanced stiffness and energy absorption capacity [32].

In recent years, the merits of graded cellular structures with varied densities or topological configurations have been shown gradually based on conventional uniform cellular materials (with a single type of cell and the same mechanical properties in all regions) [33,34,35]. By employing a TPMS-based (Triply Periodic Minimal Surface) fillet shape strategy for metallic cubic lattice architectures, Iandiorio et al. [36] realized gradient optimization inside the unit cell, leading to significant improvement in structural strength. It was found that the mechanical performance of a graded cellular structure varies with the impact process. It exhibits better energy absorption and mechanical properties than uniform cellular structures [37,38]. Based on the rigid, perfectly plastic, locking (R-PP-L) model, the classical theoretical model of dynamic compressive properties for regular cellular materials [39,40], many analytical models have been developed to reveal the excellent energy dissipation of graded cellular materials [41,42].

The graded structural concept has been successfully applied to the design of auxetic materials to achieve superior mechanical performance [43,44,45]. Qiao et al. [46] demonstrated that functionally graded double-arrowhead auxetic honeycombs exhibit better energy absorption capacity than their uniform counterparts under high-velocity impact. Liu et al. [47] reported that irregular re-entrant auxetic honeycombs possess enhanced energy absorption under quasi-static in-plane crushing compared to regular honeycombs. Xiao et al. [48] compared unidirectionally and bidirectionally graded metallic re-entrant honeycombs under compression, showing that the bidirectional grading leads to higher energy dissipation. Shao et al. [49] identified a shrinkage deformation mechanism in negatively graded auxetic honeycombs that contributes to superior energy dissipation. Novak et al. [50] found that non-graded auxetic chiral structures exhibit stiffer responses under compression and shear but fail abruptly at lower strains compared to graded designs. Further, Jiang et al. [51] systematically studied the in-plane crushing behavior of uniform, positive-gradient, and negative-gradient re-entrant circular honeycombs under various impact velocities, while Han et al. [52] showed that graded auxetic tubular structures possess higher energy absorption and stability under inclined loading.

In summary, density-graded architectures have demonstrated remarkable potential for impact resistance and energy absorption compared to uniform materials. However, fully leveraging the energy-absorption capacity of graded auxetic lattices requires careful design of the density profile. Although previous studies have explored graded auxetic honeycombs, most are limited to two-dimensional configurations or simple gradient patterns. There remains a significant lack of understanding regarding the dynamic compression behavior of 3D graded auxetic lattices, particularly how the coupling between the density gradient and the NPR affects their impact resistance and energy dissipation mechanisms. Moreover, theoretical models predicting the dynamic impact response of such graded metamaterials are still scarce.

To address this research gap, the present study designs a novel graded auxetic lattice metamaterial based on a 3D re-entrant arrowhead structure. We aim to systematically investigate its dynamic compressive response, deformation modes, and energy dissipation mechanisms. Theoretical analyses are conducted to uncover the physical origins of the enhanced energy absorption and to predict the dynamic performance of the proposed graded auxetic metamaterial.

## 2. Methods and Experiment

The dynamic impact properties of the basic auxetic lattice were first investigated using FE analysis and experiments. In this section, FE simulation and experimental methods are introduced, and a systematic comparison is performed.

### 2.1. Simulation Method

**Figure 1:**
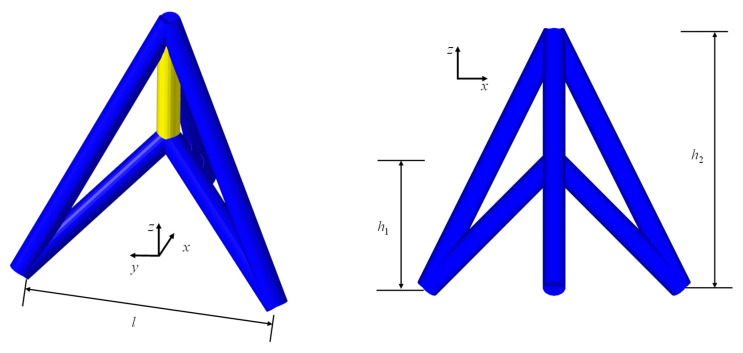
The geometric parameters of re-entrant arrowhead unit cell.

As an extension of our prior research [27], the unit cell of a 3D re-entrant auxetic lattice (Figure 1), recognized for its high stiffness and energy absorption capacity, is employed as the fundamental architecture in this study to construct auxetic lattice metamaterials. The topology of unit cells can be characterized using three geometrical parameters: height parameter *h*_1_, height parameter *h*_2,_ and length parameter *l*. In addition, a circular bar with radius *r* was adopted as the basic member of the lattice. Then, the relative density of the auxetic lattice can be derived by unit cell analysis as follows:

(1) $$\bar{\rho }=\frac{4\sqrt{3}\pi \left[\sqrt{h_{2}^{2}+l^{2}/3}+\sqrt{h_{1}^{2}+l^{2}/3}+\left(h_{2}-h_{1}\right)/3\right]r^{2}}{\left(h_{2}-h_{1}\right)l^{2}}$$

**Figure 2:**
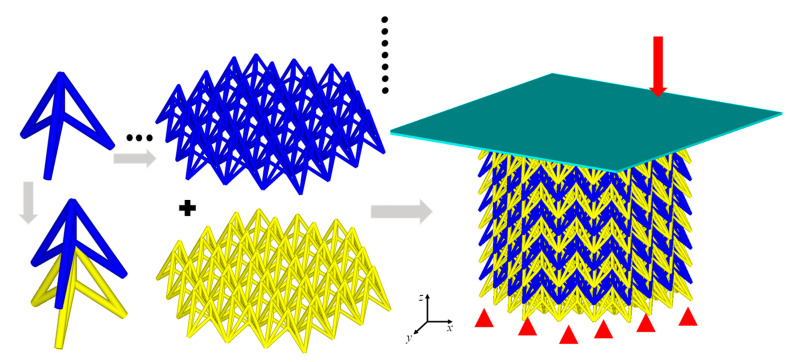
The FE model of the auxetic lattice.

By assembling the unit cells, a uniform auxetic lattice structure (42 cells per layer, nine layers, *h*_1_ = 10 mm, *l* = 20 mm, *h_2_* = 20 mm, *r* = 0.8 mm) was constructed, as shown in Figure 2. The cross-sectional projection of the overall auxetic lattice structure was a regular hexagon. The nonlinear FE package LS-DYNA R16.1.0 was employed to simulate the impact and dynamic compression of auxetic lattices.

Considering the significant computational cost associated with solid elements, we chose to simulate the lattice structure using elastic-plastic beam elements. This approach has been validated by experimental studies, demonstrating its effectiveness in accurately capturing the mechanical behavior of the lattice structure [53,54]. The auxetic lattice structure is discretized using the Belytschko-Schwer resultant beam elements in LS-DYNA, which is suitable for simulating the dynamic response of the lattice structure under complicated loads [55]. In the unit cell, beam elements with sizes of 5 mm and 3 mm were adopted for the long and short struts, respectively, ensuring that each strut contained more than three elements. The results from this beam element model demonstrated excellent agreement with those from a refined solid element submodel, not only in the macroscopic force-displacement curve but also in both the global and local deformation patterns. The auxetic lattice model, comprising 13,122 elements, was part of a full model that reached 14,144 elements after meshing the rigid impactor with shell elements. Contact interactions in the nonlinear FE simulations were governed by specific algorithms. The contact between the impactor and the auxetic lattice was defined using *CONTACT_AUTOMATIC_NODES_TO_SURFACE in LS-DYNA, which checks for penetration of slave nodes (from the lattice) into the master segments (of the rigid impactor). Self-contact within the lattice structure was modeled with *CONTACT_AUTOMATIC_GENERAL, a type suitable for simulating edge-to-edge contact among beam elements. A friction coefficient of 0.20 was assigned for both static and dynamic conditions in all contact definitions. Hourglass control and mass scaling were activated in numerical models to enhance numerical stability. Full constraints were applied at the bottom end of the auxetic structure and the compressive velocity was prescribed to the impactor. An FE simulation model of the auxetic lattice was constructed under these conditions, as illustrated in Figure 2.

In this study, 316L stainless steel was used as parent material for the lattice structure. The elastic properties of the bulk material are Young’s modulus of 200 GPa and Poisson’s ratio of 0.3. The Johnson-Cook empirical constitutive equation from the MAT-98 material model in LS-DYNA [55] was adopted to characterize the constitutive relation of the bulk material, regardless of the effect of temperature, as follows:

(2) $$\sigma =\left(A+B\varepsilon^{n}\right)\left(1+C\ln\frac{\dot{\varepsilon }}{{\dot{\varepsilon }}_{0}}\right)$$

where ε  is the plastic strain and $\dot{\varepsilon }$ denotes the strain rate. The parameters of the bulk material were set to *A* = 550 MPa, *B* = 221 MPa, *C* = 0.23, and *n* = 0.6. The parameters employed in our study are validated to be consistent with the established range for the Johnson-Cook model of 316L material found in the literature [56,57,58,59]. It is noteworthy that the referenced studies, which predominantly utilize the Johnson-Cook model for impact simulations on honeycomb or lattice structures, have yielded highly reliable results, further supporting the applicability of our parameter selection. The impactor was a rigid component of the MAT-20 material.

### 2.2. Impact Experiment

**Figure 3:**
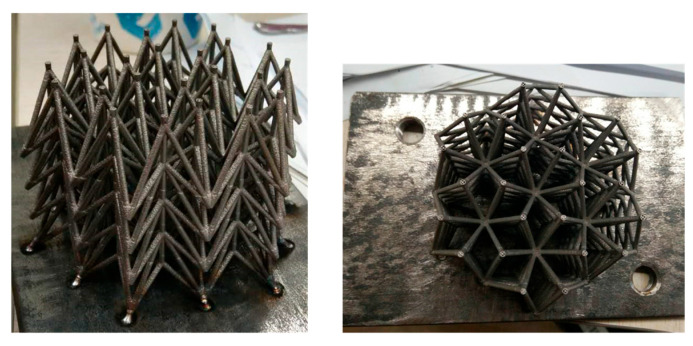
The auxetic lattice sample manufactured by SLM.

An auxetic lattice specimen (24 cells per layer, four layers, *h*_1_ = 10 mm, *l* = 20 mm, *h*_2_ = 20 mm, *r* = 0.8 mm) was manufactured using selective laser melting (SLM). This raw material of the auxetic lattice adopted 316L stainless steel powder in a spherical shape with a diameter ranging from 20 μm to 30 μm and 99.9% purity. The SLM equipment has a power of 100 W and a speed of 300 mm/s, which allows 80 μm hatch spacing and 30 μm layer thickness. The constitutive parameters of 316L stainless steel are Young’s modulus of 200 GPa, Poisson’s ratio of 0.3, yield strength of 550 MPa, and tensile strength of 654 MPa. The auxetic lattice sample printed using the SLM equipment is shown in Figure 3.

**Figure 4:**
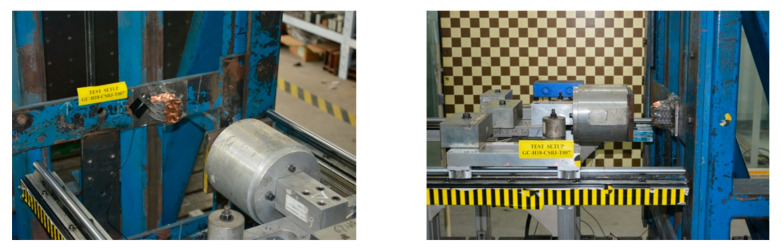
The table-impact experiment of the auxetic lattice structure.

We developed a specialized impact-testing apparatus, as depicted in Figure 4, to comprehensively evaluate the dynamic impact behavior of the auxetic lattice. The experimental setup consists of a motor (6P, power of 0.55 KW) connected to a reducer (two-stage reduction, reduction ratio of 473), which drives a spring to store and subsequently release energy. This mechanism enables the conversion of potential energy into kinetic energy in the impactor, facilitating controlled and repeatable impact experiments.

To ensure precise control and measurement of the impact velocity, we employed a compressive spring system with an impactor mass of 43 kg governed by the formula *Kx =* 1/2 *mv*^2^, where *K* represents the spring stiffness and *x* denotes the compressive length. However, owing to factors such as energy loss from track friction and uncertainties in spring stiffness, accurately determining the impact velocity based solely on these parameters is challenging. Therefore, we installed a laser speedometer at the impact position to directly measure the instantaneous velocity during the experiment, with an accuracy of 0.01 mm/ms. For this specific impact experiment, a target velocity of 7 m/s was set and the measured instantaneous velocity recorded during the test was 6.92 m/s. To capture the dynamic behavior of the impact, two acceleration sensors (Model: MPA1064A-2000, sensitivity: 142.7 μv/g, manufacturer: Beijing Lingxin Zhiyuan Technology Co., Ltd., Beijing, China) were symmetrically positioned on both sides of the impactor to record the acceleration precisely throughout the impact event. The accelerometer operates at a sampling frequency of 50 kHz, exhibiting a deviation of less than 1%. The arrangement of the impact experimental table and the sample setup are shown in Figure 4.

**Figure 5:**
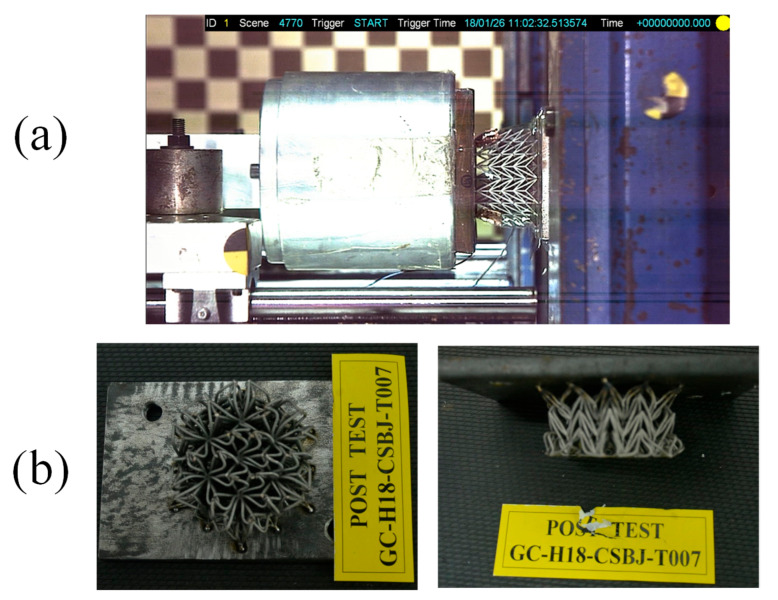
(**a**) The impact process at 0 s high-speed camera recorded; (**b**) the auxetic lattice sample after impact.

A high-speed camera (Model: NAC high-speed camera GX-3, manufacturer: NAC image technology, Salem, U.S.A.) adjacent to the specimen was positioned to provide a visual representation of the dynamic deformation process during the impact. This camera can capture the transient behavior of an impact with a high temporal resolution. Figure 5a displays the recorded image at the zero-time point of the impact process, illustrating the initial stage of the impact. Notably, the auxetic lattice sample exhibited remarkable resistance to fracture or damage, as demonstrated in Figure 5b.

### 2.3. Comparison Between Experiment and Simulation

**Figure 6:**
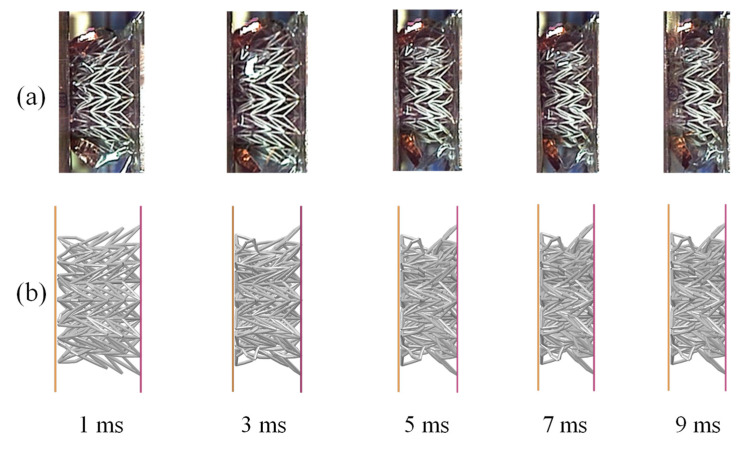
The deformation comparison between (**a**) simulation and (**b**) experiment.

An FE simulation was conducted to replicate the impact experiments under identical conditions. The numerical results demonstrate a well-balanced exchange between kinetic and internal energy, thereby ensuring the conservation of total energy. Furthermore, the hourglass energy was effectively controlled below 0.5%, which further affirms the reliability of the numerical calculations. The impact procedure in the experiment was directly recorded using a high-speed camera, allowing for a detailed comparison between the deformation responses obtained from the simulation and experiment. Figure 6 presents a comparative analysis of the deformation responses. Notably, a distinct concave phenomenon was observed during the impact process, and the timing of the deformation in both the simulation and experiment coincided remarkably well. This agreement between the simulation and experiment further validates the accuracy and reliability of the computational model for capturing the dynamic behavior of the auxetic lattice under impact conditions.

**Figure 7:**
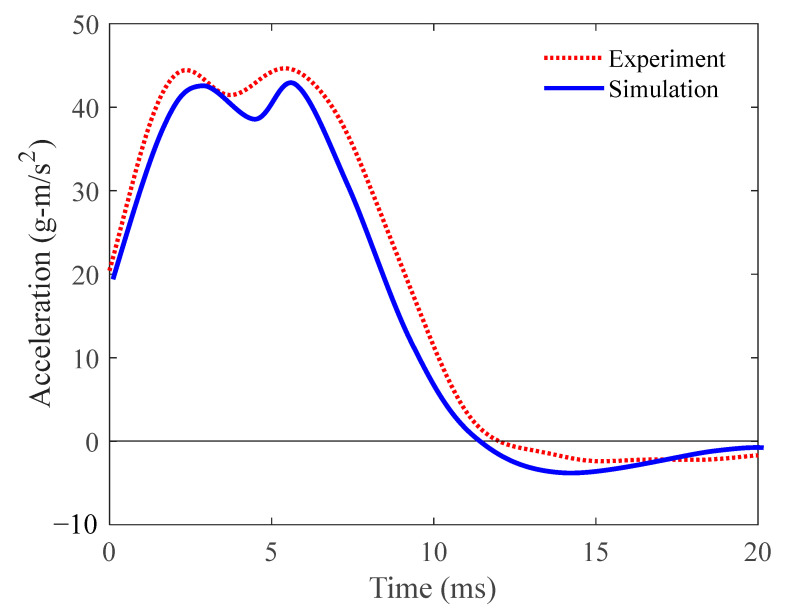
The comparison of acceleration between experiment and simulation.

In addition to the impact deformation, the impact acceleration can also be obtained from experiments and simulations. The raw acceleration data of the impact experiment were filtered using an SAE-180 (Manufacturer: Scientific Audio Electronics Inc., Los Angeles, U.S.A.) filter window and compared with the simulation data, as depicted in Figure 7. The acceleration curves of both the experiment and simulation exhibited consistent patterns characterized by double peaks. Notably, there were no excessively high acceleration peaks, indicating that the auxetic lattice possessed commendable energy absorption capabilities. The peak accelerations from the simulation and experiment differed by approximately 7%, with the simulation yielding lower results. By employing quadratic integration of the acceleration curve, the experiment calculated the maximum deformation of the lattice structure as 20.03 mm. The maximum deformation provided by the simulation was 21.25 mm. The deviation in the maximum displacement between the simulation and the experiment was 6.1%.

In conclusion, the comparative results demonstrate, to a certain extent, the validity of the current FE modeling approach in simulating the dynamic crushing behavior of the auxetic lattice structure. This validated FE model exhibits potential utility for further investigating the dynamic compression characteristics of such lattices.

## 3. Crashworthiness of Auxetic Lattices

The mechanical responses of cellular structures exhibit sensitivity to the impact velocity or strain rate [60]. To comprehensively investigate the impact mechanical properties of auxetic lattices, it is crucial to expand the range of compressive velocities. Thus, this Section aims to analyze the influence of compressive velocity on the mechanical performance of auxetic lattices by conducting compressive simulations at velocities of *v* = 1, 10, and 100 m/s, respectively.

### 3.1. Graded Auxetic Lattice Configuration

Functionally graded materials are commonly achieved by employing density-varying patterns. In this regard, the relative density of auxetic lattice materials can be conveniently adjusted by altering the cross-sectional radius of the basic bars, as indicated in Equation (1). This approach allows the customization of material properties without the need to modify the geometrical configuration of the unit cell.

**Figure 8:**
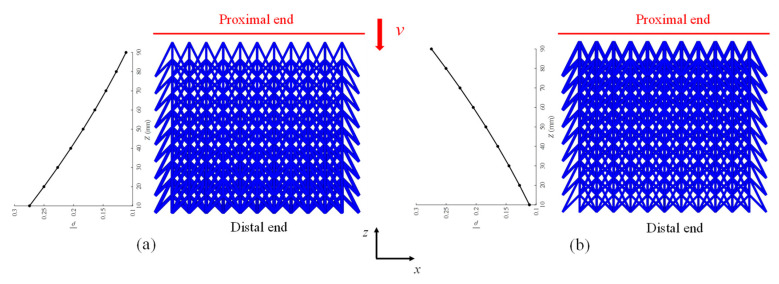
The configurations of radius-graded auxetic lattice: (**a**) gradient-A; (**b**) gradient-B.

In this section, two types of layer radius-graded auxetic lattices are proposed based on the fundamental configuration (42 cells per layer, nine layers, *h*_1_ = 10 mm, *l* = 20 mm, *h*_2_ = 20 mm). The radius of every layer follows an arithmetic progression, with a common difference of 0.05 mm between adjacent layer radii. The radii of the bars in the nine layers of the graded lattices are arranged as follows: 0.7, 0.75, 0.8, 0.85, 0.9, 0.95, 1.0, 1.05, and 1.1 mm, respectively. The auxetic lattice that exhibits a layer-by-layer ascending radius of bars from the proximal (moving) to the distal (fixed) ends is referred to as the gradient-A lattice. On the other hand, the configuration with a layer-by-layer descending radius is known as the gradient-B lattice. The two configurations are visually depicted in Figure 8.

To ensure mass consistency between the uniform auxetic lattice and the graded auxetic lattices (1.248 kg), the radius of each strut in the uniform lattice was set to 0.91 mm. With this configuration, the numerically calculated mass of the uniform auxetic lattice was 1.2507 kg, deviating by only 0.2% from that of the graded structures. This radius adjustment effectively maintains equivalent mass across the uniform and graded designs. Furthermore, the effective density of the overall structure was calculated as 1334.3 kg/m^3^, corresponding to a relative density of 16.89% for both graded auxetic lattices. For the uniform auxetic lattice, the effective density was 1337.2 kg/m^3^, yielding a relative density of 16.92%.

### 3.2. Criteria for Crashworthiness Evaluation

Several critical performance criteria were selected to evaluate the compressive properties of the auxetic lattices. The impact energy absorbed by the material can be dissipated through the elastic-plastic deformation of the bars in lattice structures. Specific energy absorption (SEA) is used to characterize the absorbed impact energy per mass, which can be calculated as

(3) $$\mathrm{SEA}=\frac{\int_{0}^{D_{a}}FdD}{M}$$

where *D*_a_ denotes the current deformation, *F* represents the compressive force of the indenter, and *M* denotes the mass of the structure. At a given equivalent strain or deformation level, the SEA can effectively evaluate the impact energy absorption capacity of the structure. To quantify the mechanical properties of the auxetic lattice material, the nominal stress and strain are defined as follows:

(4) $$\begin{array}{l} \sigma =\frac{F}{A}\\ \varepsilon =\frac{D_{a}}{L} \end{array}$$

where *A* represents the projected area of the lattice structure, and *L* denotes the length between the two ends of the structure along the compression direction (*z*-direction). By utilizing Equation (4), the nominal stress–strain curve can be obtained from the impact force-deformation curve in the simulation or experiment. In the nominal stress–strain curve, the initial peak stress *σ*_m_ serves as an essential indicator for evaluating the energy-absorption performance. A large initial peak stress is not desirable for crashworthiness because it can lead to excessive acceleration of the protected structure.

The energy-absorption capability of structures or materials is commonly characterized by an ideal energy-absorption efficiency *η*. Additionally, parameter *η* [61] is determined by

(5) $$\eta =\frac{\int_{0}^{\varepsilon_{a}}\sigma d\varepsilon }{\sigma_{m}\varepsilon_{a}}$$

where *ε*_a_ is the current strain and *σ*_m_ denotes the initial peak stress. Furthermore, the definition of energy efficiency *ω* is given as

(6) $$\omega =\frac{\int_{0}^{\varepsilon_{a}}\sigma d\varepsilon }{\sigma_{a}}$$

The representative densification strain, denoted as *ε*_d_, can be determined by identifying the point on the energy efficiency-strain curve where the energy efficiency reaches its global maximum [61].

Plateau stress is another significant indicator for evaluating the energy absorption capability of lattice structures. The plateau region starts from the crushing strain *ε*_m_ where the stress reaches the first peak and ends at the densification strain *ε*_d_. The plateau stress *ε*_pl_ refers to the average stress in the plateau region, which is determined by:

(7) $$\sigma_{\mathrm{pl}}=\frac{1}{\varepsilon_{d}-\varepsilon_{m}}\int_{\varepsilon_{m}}^{\varepsilon_{d}}\sigma d\varepsilon$$

### 3.3. Dynamic Compression of Auxetic Lattices

#### 3.3.1. Compressive Velocity of 1 m/s

**Figure 9:**
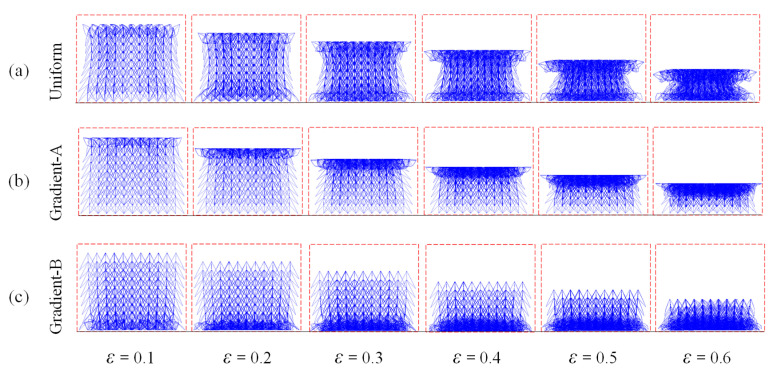
The compressive deformation sequences of three auxetic lattices at *xz*-plane under the velocity of 1 m/s: (**a**) Uniform lattice; (**b**) Gradient-A lattice; (**c**) Gradient-B lattice.

Compressive simulations at a constant velocity of 1 m/s were first conducted for three auxetic lattices, the deformation stages of which are shown in Figure 9. The deformations of the uniform lattice exhibit shapes of “> <“ caused by the bending and buckling of bars, indicating an obvious NPR appearance in the whole structure. During the compression process, the top and bottom parts of the uniform structure collapsed simultaneously. However, the graded lattices exhibited a layer-by-layer deformation pattern, and the transverse shrinkage effect appeared only in the densified part. For the gradient-A lattice, collapse occurs first at the proximal end with the lowest density. When the low-density parts of the graded lattice are placed at the distal end as the gradient-B lattice, crushing first occurs at the support end, as depicted in Figure 9c.

**Figure 10:**
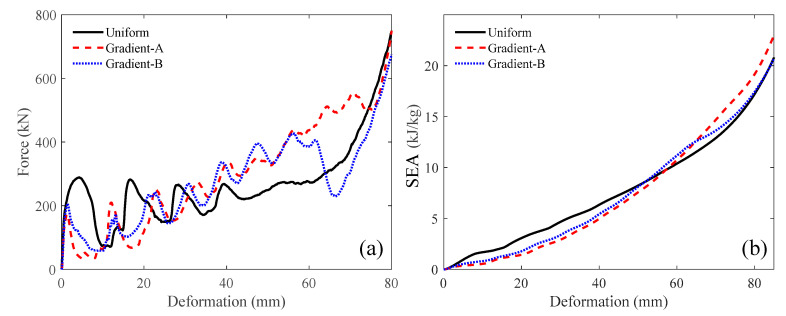
The compressive performances at the velocity of 1 m/s: (**a**) force-deformation; (**b**) SEA.

**Figure 11:**
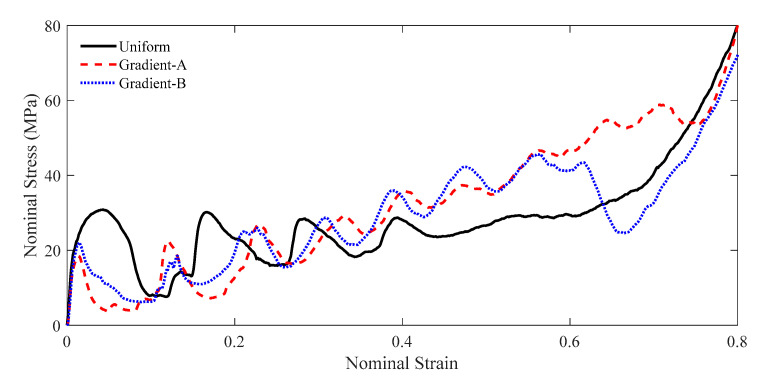
The nominal stress–strain curve at the compressive velocity of 1 m/s.

**Figure 12:**
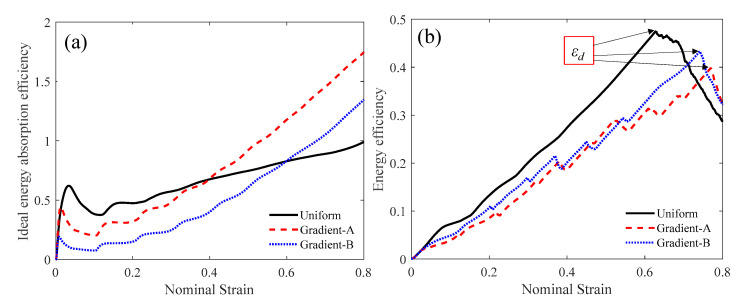
The compressive performances at the velocity of 1 m/s: (**a**) ideal energy absorption efficiency; (**b**) energy efficiency.

The force-deformation curve can be directly obtained from the simulation, as shown in Figure 10. The specific energy absorption can then be calculated using Equation (3), and their curves are depicted in Figure 10b. At the later stage of compression, the gradient-A lattice achieved the largest SEA. The nominal stress–strain curve can be obtained from the force-deformation curve through Equation (4), as shown in Figure 11. Under a low velocity, the uniform lattice had the largest initial peak stress and a noticeable plateau stress period. The gradient-A and gradient-B lattices exhibited step-wise stress fluctuations and gradually increased the stress magnitude. Moreover, the ideal energy absorption efficiency curve can be derived through Equation (5), as illustrated in Figure 12a. The graded lattices had a larger *η* than the uniform lattice at the later stage. The energy efficiency curve can then be used to capture the densification strain, as shown in Figure 12b.

The crashworthiness indicators of the three auxetic lattices can be extracted and are listed in Table 1. When the strain was 0.8, the gradient-B lattice had an approximate SEA with the uniform lattice, and the gradient-A lattice had the largest SEA. Compared to the uniform lattice, the initial peak stress *σ*_m_ of the gradient-A lattice was reduced by 40.1%. The gradient-A lattice had the largest plateau stress *σ*_pl_, which was 44.43% higher than that of the uniform lattice. The densification strain *ε*_d_ of the two graded lattices was larger than that of the uniform lattice.

#### 3.3.2. Compressive Velocity of 10 m/s

**Figure 13:**
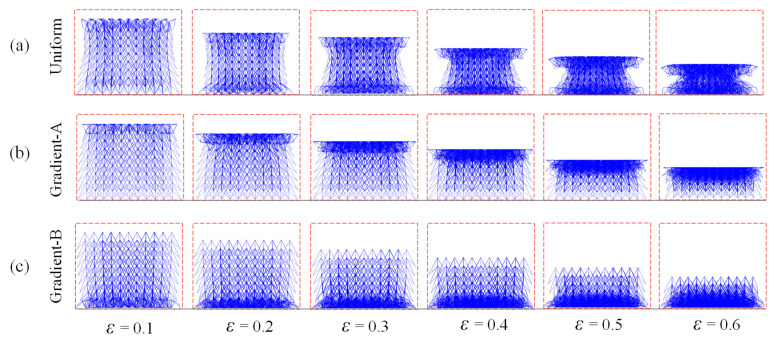
The compressive deformation sequences of three auxetic lattices at *xz*-plane under the velocity of 10 m/s: (**a**) Uniform lattice; (**b**) Gradient-A lattice; (**c**) Gradient-B lattice.

**Figure 14:**
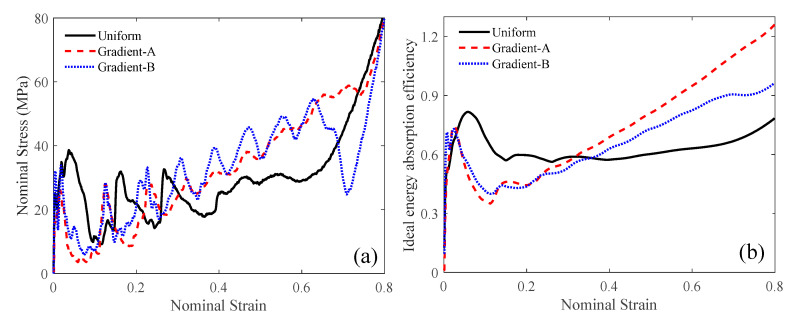
The compressive performance at the velocity of 10 m/s: (**a**) nominal strain-stress; (**b**) ideal energy absorption efficiency.

The deformations of the three auxetic lattices at a compressive velocity of 10 m/s are shown in Figure 13. The uniform lattice exhibited obvious overall shrinkage, and the graded lattices showed layer-by-layer collapse, similar to the case of 1 m/s, as illustrated in Figure 9. Under an impact of 10 m/s, the crushing pattern of the auxetic lattices hardly changed. The nominal stress–strain and ideal energy absorption efficiency can be obtained from the numerical simulations, as shown in Figure 14. The nominal stress–strain curves shown in Figure 14a do not change significantly, except for an increase in the initial peak stress compared with that at a velocity of 1 m/s, as illustrated in Figure 11. The gradient-A lattice had the largest ideal energy absorption efficiency when the strain exceeded 0.32, as shown in Figure 14b.

The indices of the compressive properties at 10 m/s are listed in Table 2. Compared to Table 1, *σ*_m_ values of the three auxetic lattices increased noticeably. However, the gradient-A lattice still had the largest SEA and the smallest *σ*_m_ at 10 m/s.

#### 3.3.3. Compressive Velocity of 100 m/s

**Figure 15:**
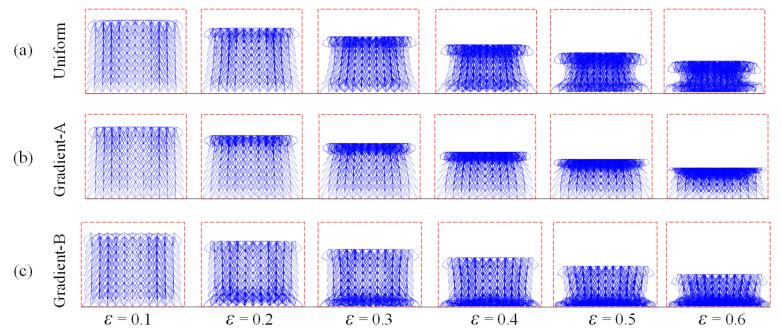
The compressive deformation sequences of three auxetic lattices at *xz*-plane under the velocity of 100 m/s: (**a**) Uniform lattice; (**b**) Gradient-A lattice; (**c**) Gradient-B lattice.

Nonetheless, the top parts of the three lattices crush earlier than the bottom parts at the high velocity of 100 m/s, as depicted in Figure 15. Thus, at a strain of 0.4, the uniform lattice exhibited a ladder-like deformation, as illustrated in Figure 15a. Even under high-velocity impact, the deformation of the gradient-A lattice displays a layer-by-layer mode similar to that observed under low-velocity impact. For the gradient-B lattice, when the strain was 0.1, the upper part collapsed first under a high compressive velocity. Shock wave theory can reasonably explain these phenomena. At a low impact velocity, the front end deformed slowly, and the shock wave passed down to the bottom simultaneously. However, at a high impact velocity, the deformation localization time of the proximal end was extremely short, leading to a delay in the deformation of the bottom part. This results in a hysteresis effect in the bottom deformation.

**Figure 16:**
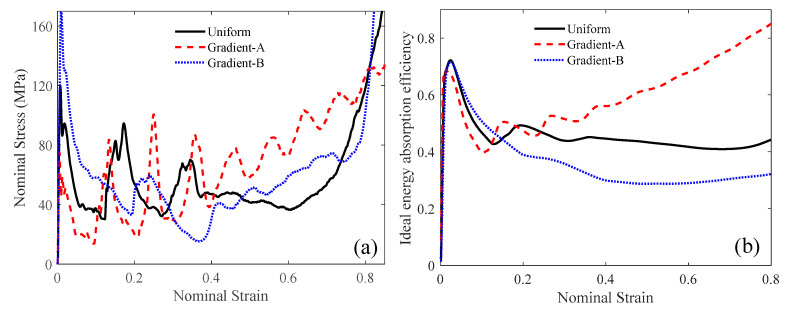
The compressive performance at the velocity of 100 m/s: (**a**) nominal strain-stress; (**b**) ideal energy absorption efficiency.

As shown in Figure 16a, the initial peak stress of the uniform and gradient-B lattices is greater than that of the gradient-A lattice. At the later compression stage, the uniform and gradient-B lattices showed similar densification patterns caused by the NPR effect. However, the gradient-A lattice still had nine stress cycles, implying layer-by-layer crushing. The gradient-A lattice had the largest energy absorption efficiency when the strain exceeded 0.25, as shown in Figure 16b. In Table 3, the gradient-A lattice had the largest SEA of 41.22 kJ/kg. The initial peak stress *σ*_m_ of the gradient-A lattice decreased by 37.49% compared to that of the uniform lattice and by 55.77% compared to that of the gradient-B lattice.

Based on the analysis results obtained from the FE simulations, it can be concluded that both the gradient profile and compressive velocity play a substantial role in influencing the impact mechanical performance of auxetic lattices. In particular, the gradient-A lattice demonstrates superior crashworthiness characteristics compared to the other lattices. By employing the gradient-A configuration, the initial peak stress can be significantly reduced while simultaneously enhancing the energy absorption capability. Therefore, the gradient-A configuration is deemed more favorable for achieving improved crashworthiness in auxetic lattice design.

## 4. Discussion

In this section, we develop semi-theoretical models to reveal the energy absorption mechanism and conduct a parametric analysis of the graded auxetic lattice metamaterial.

### 4.1. Theoretical Analysis Models

#### 4.1.1. Quasi-Static Compression

In Section 3, a functionally graded auxetic structure is created by altering the radius of the bars between the rows. The analytical expressions for the effective Young’s modulus and yield stress of the uniform auxetic lattice were initially derived as follows.

**Figure 17:**
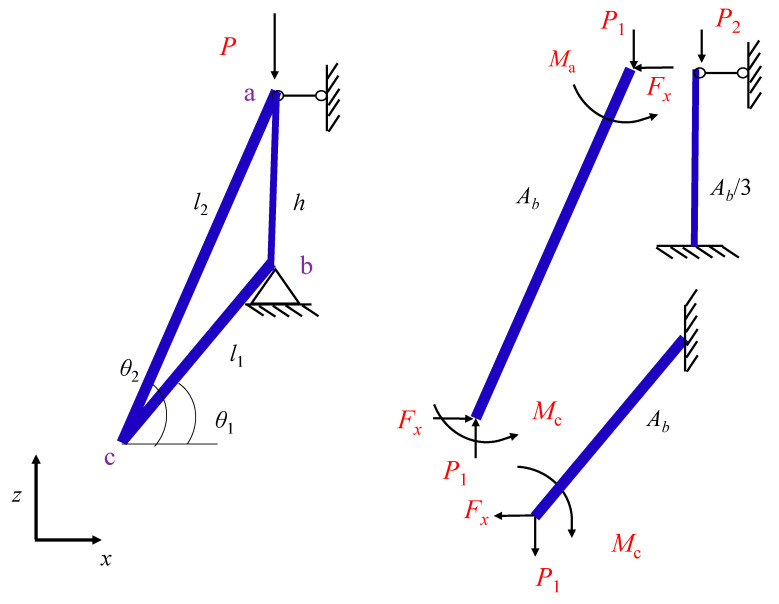
One-third analytical model of unit cell.

To facilitate the analytical derivation of the unit cell, the one-third model shown in Figure 17 was considered because of the geometrical symmetry of the auxetic structure. The use of the Euler-Bernoulli beam theory allows for theoretical calculations because the structure is composed of slender bars. The parameters *l*_1_, *l*_2_, *h*, sectional area *A_b_*, *θ*_1_, *θ*_2_ can be expressed in relation to the base geometrical parameters of the unit cell defined in Figure 1. The components of the force and moment in the model can be expressed in terms of the vertical force, as *P*_1_ = *C*_1_*P, P*_2_ = (1 − *C*_1_)*P*, *F_x_* = *C_F_P*_1_, *M*_a_ = *C_M_*_a_*P*_1_, and *M*_c_ = *C_M_*_c_*P*_1_, solved by applying Castigliano’s second theorem. The detailed derivations are provided in Appendix A.

In Figure 17, the total vertical force is

(8) $$P=P_{1}+P_{2}=\sigma A_{u}/3$$

where *A*_u_ is the projected area of the unit cell, and *σ* is the effective stress. For a lattice with steel as the base material, the critical force of bar-ac or -bc is dominated by the yield criterion rather than buckling. Therefore, the maximum axial stress is considered. For bar-ac, the maximum axial stress can be expressed as:

(9) $$\sigma_{\max}=\frac{P_{1}\sin\theta_{2}+F_{x}\cos\theta_{2}}{A_{b}}+\frac{M_{\max}r}{I}$$

where *M*_max_ represents the maximum moment in bar-ac, and *I* denotes the moment of inertia of an area, which can be calculated by *I* = *πr*^4^/64 for a circular section. For bar-bc, the maximum axial stress is

(10) $$\sigma_{\max}=\frac{P_{1}\sin\theta_{1}+F_{x}\cos\theta_{1}}{A_{b}}+\frac{M_{\max}r}{I}$$

Considering that the bar is slender, the stress contribution of the axial compression in the cross-section can be ignored compared to the bending. The maximum stress in strut-ac + bc can then be approximately expressed as

(11) $$\sigma_{\max}=\frac{M_{\max}r}{I}$$

When the maximum stress approaches the yield stress of the constitutive material, the allowable bending moment can be obtained. The maximum bending moment may occur along points-a, -b, or -c. Therefore, the maximum bending can be written as

(12) $$M_{\max}=\max\left\{\left|M_{a}\right|,\left|M_{c}\right|,\left|M_{a}+F_{x}h\right|\right\}$$

The vertical critical force *P*_1_ can be calculated using Equation (12). The total crushing force *P*_cr1_ can then be obtained using *P*_cr1_ = *P*_1_/*C*_1_.

For bar-ab, the critical force is dominated by compression yield or buckling. For steel as the parent material, the critical force is dominated by buckling and is calculated as follows:

(13) $$p_{2}=\frac{1}{3}\frac{\pi^{2}EI}{{\left(0.7h\right)}^{2}}$$

where *E* is Young’s modulus of the base material. Then, the total crushing force *P*_cr2_ can be calculated as *P*_cr2_ = *P*_2_/(1 − *C*_1_).

The accurate critical force *P*_cr_ of struts-ab + bc + ac is determined by the smaller value between *P*_cr1_ and *P*_cr2_. When the base material was steel, and the configuration in Figure 17 was adopted, the total crushing force was *P*_cr1_ dominated by the bending yield of the bars in strut-ac + bc. Therefore, the critical strength *σ*_cr_ can be expressed as:

(14) $$\sigma_{\mathrm{cr}}=\frac{P_{\mathrm{cr}}}{A_{u}/3}\propto \frac{\sigma_{y}I}{r}\propto \;\;r^{3}\;or\;{\bar{\rho }}^{1.5}$$

where *σ*_y_ represents the yield stress of the constitutive material.

In the quasi-static condition, the influence of inertia can be neglected. Therefore, it is reasonable for the uniform lattice structure to assume that

(15) $$\sigma_{\mathrm{cr}}^{q}=\sigma_{m}^{q}=\sigma_{\mathrm{pl}}^{q}=a{\bar{\rho }}^{1.5}$$

where the superscript ‘q’ denotes the quasi-static condition. Theoretically, the critical stress can be considered as the initial peak stress $\sigma_{m}^{q}$ or plateau stress $\sigma_{\mathrm{pl}}^{q}$ in the ideal stress–strain curve of lattice structures.

As *C*_1_ is extremely small compared to *C*_2_, the effective Young’s modulus is approximately derived as

(16) $$\bar{E}=\frac{4A_{b}}{\sqrt{3}l^{2}}E$$

Thus, the theoretical yield strain of the auxetic unit cell can be calculated by

(17) $$\varepsilon_{\mathrm{cr}}^{q}=\sigma_{\mathrm{cr}}^{q}/\bar{E}$$

The effective initial Poisson’s ratio can be calculated using Equation (A.16) in Appendix A, which is not related to the radius of the bar, but to the geometry of the unit cell.

**Figure 18:**
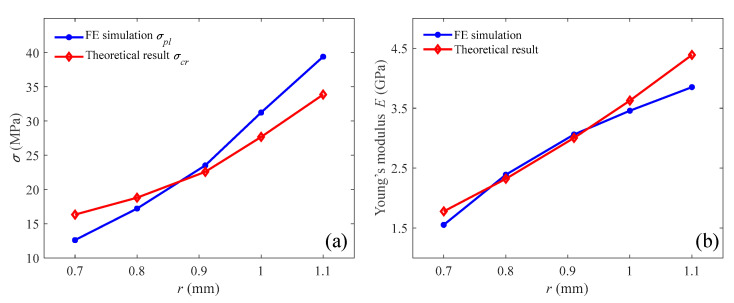
Comparison between the FE simulation and theoretical analysis for uniform lattice: (**a**) critical stress; (**b**) effective Young’s modulus.

The quasi-static compressions of the uniform auxetic lattices with radii of 0.7, 0.8, 0.91, 1.0, and 1.1 mm were evaluated using both FE simulations and theoretical analysis. The comparison of their critical stresses and effective Young’s moduli is shown in Figure 18. Remarkably, the results obtained from the theoretical analysis were consistent with those obtained from the FE simulations. This consistency between the two approaches signifies the validity and reliability of the theoretical analysis model for predicting the mechanical behavior of uniform auxetic lattices.

For the graded auxetic lattice, the elastic modulus and initial crushing stress in the quasi-static condition are determined by the layer with the smallest density *ρ*_min_. As the plateau stress increases with the relative density, the graded lattice has increasingly step-wise stress in the plateau region of the stress–strain curve, as shown in Figure 11. For a monotonically varying gradient lattice, we assume that the stress–strain curve conforms to the monotonic linear increasing law with slope *k*

(18) $$k=\frac{\sigma_{d}-\sigma_{m}}{\varepsilon_{d}-\varepsilon_{m}}$$

For a graded lattice material, the slope *k_g_* caused by the density-gradient configuration can be expressed as follows:

(19) $$k_{g}=a\frac{{\bar{\rho }}_{\max}^{1.5}-{\bar{\rho }}_{\min}^{1.5}}{\varepsilon_{d}-\varepsilon_{m}}$$

**Figure 19:**
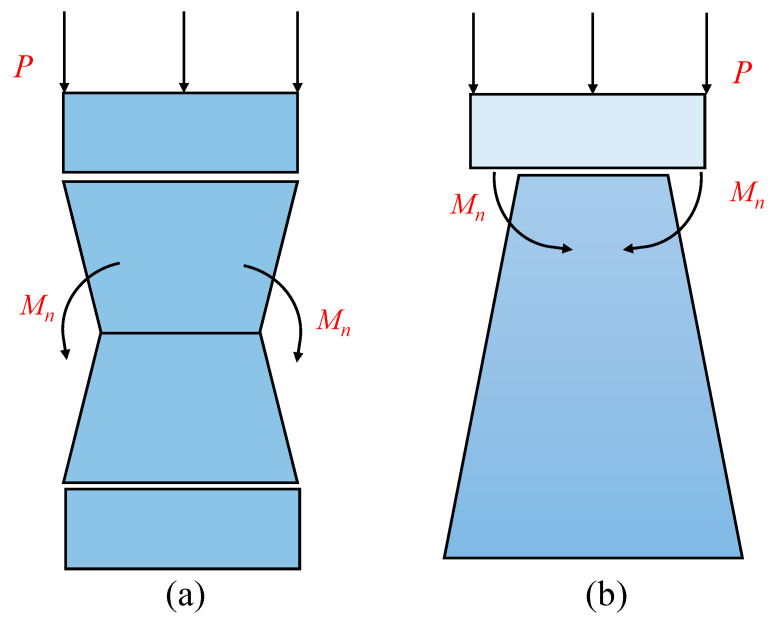
Schemes of the force states for the (**a**) uniform lattice and (**b**) gradient-A lattice.

For a uniform auxetic lattice, the additional bending moment can promote the shrinkage of the deformation toward the center of the auxetic lattice, which can be balanced by the crushing force [49], as shown in Figure 19a. However, when the shrinkage occurs at the densification position for the graded auxetic lattice, the additional bending moment could enhance the crushing force, as illustrated in Figure 19b. For the graded auxetic material, we assume that the coupling effect of the graded configuration and NPR is simplified by the combined slope of the plateau stress, which can be rewritten as

(20) $$k=k_{g}k_{n}$$

where *k*_n_ is the enhanced factor caused by the NPR effect. By utilizing Equation (19) and the data presented in Figure 18, *k*_g_ of the graded lattices was calculated as 26.984 MPa. Furthermore, based on the simulation data in Section 3.3.1, the slope of the gradient-A lattice calculated using Equation (18), was determined to be *k* = 67.562 MPa. Therefore, the semi-empirical *k*_n_ of the graded auxetic lattice is ~2.5. It can be observed from Equation (A16) that Poisson’s ratio is independent of the radius of the bars and is solely influenced by the topological geometric parameters of the structure. Therefore, in this gradient configuration, it is assumed that the *k*_n_ value remains constant.

In the graded auxetic lattice, we also used the plateau stress (average stress) to characterize the intermediate field of the stress curve. Considering the slope field of the graded auxetic lattice structure, the plateau stress of the graded auxetic lattice is

(21) $$\sigma_{\mathrm{pl}}=\sigma_{m}+\frac{\left(\varepsilon_{d}-\varepsilon_{m}\right)k}{2}$$

where *σ*_m_ represents the crushing stress of the layer with the lowest relative density. The plateau stress is determined by the slope of the stress curve, which can be used to evaluate the energy absorption capability.

As the crushing stress in Equation (15) is a concave function, it is worth noting that

(22) $$\sigma_{\mathrm{pl}}=\frac{a}{9}\sum_{i=1}^{9}{\bar{\rho }}_{i}^{1.5}>a{\left(\frac{1}{9}\sum_{i=1}^{9}{\bar{\rho }}_{i}\right)}^{1.5}=a{\bar{\rho }}_{0}^{1.5}$$

where the left term of the inequality is the idealized plateau stress of the graded lattices and the right term is that of the uniform lattice with the same effective relative density ${\bar{\rho }}_{0}$. This can partly explain why the plateau stress of the gradient lattices is higher than that of the uniform lattice with the same relative density, as described in Section 3. Moreover, owing to the enhanced factor *k*_n_ caused by the NPR effect, the plateau stress of graded auxetic lattices can be further improved.

**Figure 20:**
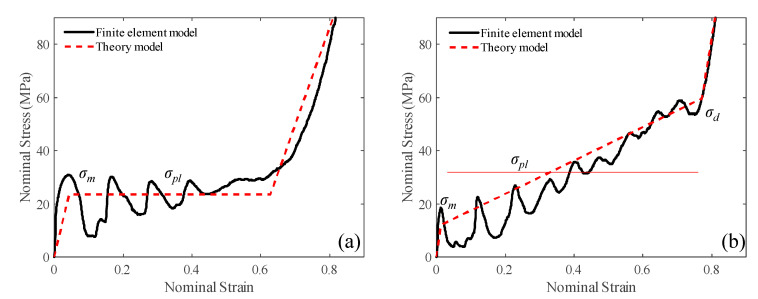
The stress–strain curves of the theoretical model under quasi-static condition: (**a**) uniform lattice; (**b**) gradient-A lattice.

The simplified analytical models we proposed successfully capture the key characteristics of the nominal stress–strain curves for the uniform and gradient-A auxetic lattices in Section 3, as depicted in Figure 20. However, it should be noted that the classic R-PP-L theoretical model only accounts for the plateau region of the stress–strain curve. In contrast, our analytical models consider the elastic field, initial crushing stress, and gradient area, allowing us to effectively describe the compressive properties of graded auxetic lattices, particularly in terms of the stress slope and the enhancement resulting from the NPR effect.

#### 4.1.2. Dynamic Compression

**Figure 21:**
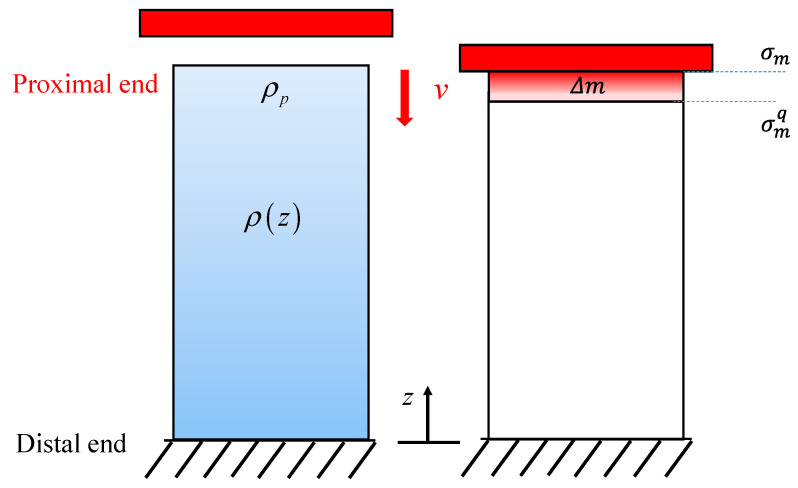
The analytical model of dynamic compression.

However, it is crucial to consider the inertial effect when dealing with high-impact or compressive velocities. In such scenarios, the influence of inertia becomes significant, leading to a substantial increase in the initial peak stress compared with the quasi-static condition. In the case of a shock front generated by dynamic compression, as depicted in Figure 21, the critical stress at high velocities is governed by:

(23) $$\left(\sigma_{m}-\sigma_{m}^{q}\right)A\delta t=\Delta mv$$

where Δ*m* is the mass of the proximal end, which can be approximately expressed as:

(24) $$\Delta m=\rho_{p}AD_{a}=\rho_{p}Ac_{p}\delta t$$

where *ρ*_p_ denotes the density of the local structure at the impacting end, and *c*_p_ is the velocity of the shock front, calculated as:

(25) $$c_{p}=\sqrt{\bar{E}/\rho_{p}}$$

here $\bar{E}$ is the effective Young’s modulus of the local structure given in Equation (16). Notably, the simplification of ignoring local contact compaction at the impact end in this study may lead to an underestimation of the effective elastic modulus in Equation (16), resulting in a lower predicted shock front velocity. By combining Equation (15) with Equations (23) and (24), we obtain the crushing strength at a high velocity as:

(26) $$\sigma_{m}=\sigma_{m}^{q}+\rho_{p}c_{p}v=a{\bar{\rho }}_{p}^{1.5}+v\sqrt{\bar{E}{\bar{\rho }}_{p}\rho_{0}}$$

where ${\bar{\rho }}_{p}$ denotes the relative density of the proximal end. Equation (26) indicates that the initial peak stress is highly dependent on the relative density of the proximal end and compressive velocity.

The inertia effect dominates the impact performance at high velocity, which leads to deformation localization and strength enhancement, as observed by Reid and Peng (1997) [39] in their direct impact tests on wood. They proposed the R-PP-L model and employed it to simplify the plateau stress of the cellular material under dynamic impact conditions, resulting in the following formulation:

(27) $$\sigma_{\mathrm{pl}}=\sigma_{\mathrm{pl}}^{q}+\frac{\rho v^{2}}{\varepsilon_{d}}$$

where the second term is caused by inertia effects, and *ρ* represents the density of the cellular material. The plateau stress of the uniform auxetic lattice under quasi-static conditions can be described by a power-law relationship with respect to the relative density, as indicated in Equation (15). Consequently, the expression for the plateau stress of the uniform lattice in relation to the impact velocity can be reformulated as follows:

(28) $$\sigma_{\mathrm{pl}}=a{\bar{\rho }}^{1.5}+\frac{\bar{\rho }\rho_{0}v^{2}}{\varepsilon_{d}}$$

where *ρ*_0_ represents the density of bulk material. For the uniform auxetic lattice subjected to a compressive velocity of 100 m/s, the contribution of the inertia term to the crushing stress can be derived, as described in Equation (26), which is larger than that of the plateau stress, as given by Equation (28). Consequently, the initial peak stress experienced by the uniform auxetic lattice is greater than the plateau stress, as illustrated in Figure 16a and Table 3.

In the case of a graded auxetic lattice configuration subjected to dynamic impact, the densification stress *σ*_d_ is not determined solely by the maximum relative density. Instead, it is further augmented by the combined effects of NPR and the influence of inertia. This can be expressed as follows.

(29) $$\sigma_{d}=k_{n}\left(a{\bar{\rho }}_{\max}^{1.5}+\frac{{\bar{\rho }}_{\max}\rho_{0}v^{2}}{\varepsilon_{d}}\right)$$

By utilizing Equation (26), the initial peak stress of the graded auxetic lattice can be determined. Furthermore, to characterize the high-velocity compressive behavior of the auxetic lattice with a monotonically varying gradient, we also make the assumption that the stress–strain curve follows a linear increasing trend with a slope *k* calculated as

(30) $$k=\frac{k_{n}\left(a{\bar{\rho }}_{\max}^{1.5}+{\bar{\rho }}_{\max}\rho_{0}v^{2}/\varepsilon_{d}\right)-a{\bar{\rho }}_{p}^{1.5}-v\sqrt{\bar{E}{\bar{\rho }}_{p}\rho_{0}}}{\varepsilon_{d}-\varepsilon_{m}}$$

By employing Equation (30), the slope *k* of the stress can be determined to characterize the impact performance of the graded auxetic lattice. When there is an increase in the radius- or density-gradient, the slope in Equation (20) becomes larger under quasi-static conditions. At high impact velocities, the densification stress term in Equation (30) is additionally enhanced by the inertia effect, leading to a notable increase in the stress slope.

### 4.2. Parametric Analysis

In this section, we conducted a parametric analysis to investigate the influence of the radius-gradient on the compressive properties of the graded auxetic lattice, focusing on the gradient-A configuration.

As discussed in Section 3.3, we previously examined the graded auxetic lattice with a constant radius difference of Δ*r* = 0.05 mm between each layer. In this section, we further explored the effects of Δ*r* by considering values of 0.025, 0.075, and 0.1 mm for the gradient-A auxetic lattices. To maintain the same mass or effective relative density, the following equation was applied to generate the radius of the layer at the proximal end:

(31) $$\sum_{j=0}^{8}{\left(r_{0}+j\Delta r\right)}^{2}=9\times 0.91^{2}$$

**Figure 22:**
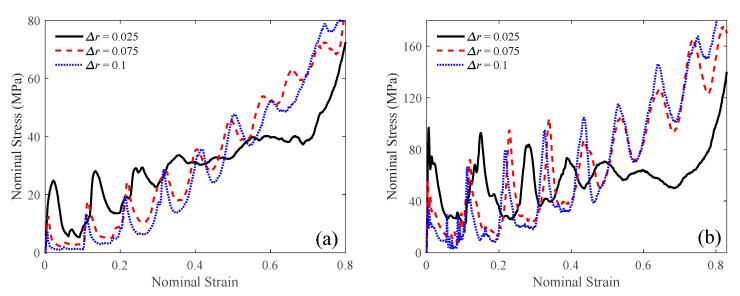
Stress–strain curves of linearly radius-graded lattices at a compressive velocity of (**a**) 1 m/s; (**b**) 100 m/s.

Subsequently, the base radii for the three auxetic lattice structures were calculated to be 0.807, 0.588, and 0.472 mm, respectively, and the corresponding sequences of the layer radii were determined. Compressive simulations under velocities of 1 and 100 m/s were performed for the three new auxetic lattices. Their nominal stress–strain curves can be obtained through an FE simulation, as depicted in Figure 22. Notably, the stress–strain curves exhibited a steeper inclination as the radius gradient Δ*r* increased.

**Figure 23:**
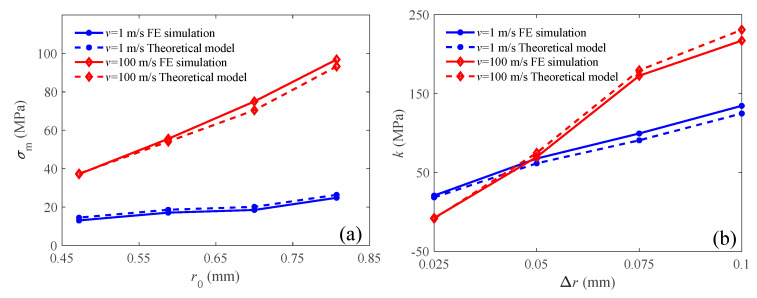
Parametric analysis of graded auxetic lattice: (**a**) initial peak stress; (**b**) slope of plateau stress.

By integrating the simulation data obtained in Section 3.3, where Δ*r* = 0.05 mm, with the current simulation results, the numerical values of initial peak stress *σ*_m_ and stress slope *k* are presented in Figure 23. Furthermore, we employed the theoretical models described in Section 4.1 to conduct the parametric analysis, comparing their results with those obtained from the FE simulations. The notable agreement between the theoretical and simulation data confirmed the validity and reliability of the proposed theoretical approach. This indicates that the theoretical models successfully captured the essential characteristics of the graded auxetic lattice’s compressive behavior, as observed in the FE simulation results. However, a notable discrepancy emerges under high-velocity impact: with an increasing basic radius, the simulated peak stress progressively exceeds the theoretical prediction. This trend is elucidated by Equation (25). The rise in relative density induces local contact compaction at the impact end causes the effective elastic modulus from Equation (16) to be underestimated, leading to a lower computed shock front velocity and a subsequently underpredicted inertial term. As a final consequence, the theoretical peak stress is calculated to be lower than the simulation value. Under high-velocity conditions, as the gradient of the inter-layer strut radius increases, the simulated stress slope gradually becomes smaller than the predicted value. This discrepancy arises primarily because the inertial term of the compaction stress in Equation (30) increases rapidly with impact velocity. When the structural relative density gradient is significant, gradient-induced deformation becomes more dominant than deformation caused by the NPR effect, resulting in a weakened coupling effect. However, Equation (30) neglects this factor for simplicity, consequently leading to an overestimation of the compaction stress.

As shown in Figure 23a, the initial peak stress *σ*_m_ was predominantly determined by the basic radius *r*_0_, which is also the radius of the layer at the proximal end. At a low velocity, the radius *r*_0_ of the impinging layer slightly affected the initial peak stress *σ*_m_. However, the crushing stress *σ*_m_ increases considerably with an increase in the base radius *r*_0_. The inertia term of the crushing stress given by Equation (26), is significantly influenced by the impact velocity and becomes the dominant contribution at high velocities.

The slope *k* of the plateau stress in the strain-stress curves can be calculated through the basic definition Equation (18). The theoretical value of slope *k* can be obtained using Equations (20) and (30), respectively. As illustrated in Figure 23b, the stress slope *k* was determined by the radius gradient Δ*r*. As Δ*r* increased, the slopes increased significantly at both the compressive velocities. When Δ*r* = 0.05, the slopes at both velocities approached approximately equal. When Δ*r* exceeded 0.05, the graded auxetic lattice under *v* = 100 m/s had a larger stress slope, indicating that the slope field had an enhanced inertia effect.

## 5. Conclusions

In conclusion, this study was designed to address a significant gap in the literature concerning the dynamic compressive behavior of three-dimensional (3D) graded auxetic lattice metamaterials. While previous research has largely focused on graded auxetic honeycombs, the coupling mechanism between the density gradient and the negative Poisson’s ratio (NPR) effect in 3D architectures under impact loading remains poorly understood. This work provides novel insights into this coupling effect through a combined approach of finite element (FE) simulation and theoretical modeling.

Our findings demonstrate that the proposed 3D re-entrant arrowhead lattice, when engineered with a positive density gradient along the loading direction, exhibits a deformation localization mechanism at the proximal end. This mechanism results in a superior energy absorption capacity compared to its uniform counterpart. Critically, the initial peak stress shows a distinct dependence on the density and impact velocity: it is governed by the lowest density region under low-velocity compression, but by the density at the proximal end under high-velocity impact.

A key theoretical contribution of this work is the development of a simplified analytical model that successfully captures the dynamic crushing stress, which is strongly dependent on impact velocity, and the unique rising stress–strain response, which is significantly influenced by both the density gradient and the NPR effect. The model reveals that the enhanced energy absorption, particularly at high velocities, stems from the synergistic coupling between the graded configuration and the NPR-induced deformation, rather than from either factor alone.

The primary limitations of the present theoretical model lie in the assumption of a contact deformation-independent effective modulus, the simplified treatment of the coupling between the density gradient and the NPR effect, and its reliance on initial FE simulation data, which results in a semi-empirical framework.

Overall, this research not only validates the superior performance of graded auxetic lattices for impact protection but also delivers a predictive theoretical framework that can guide the future design of advanced auxetic metamaterials. Future work will focus on experimental validation under a wider range of impact conditions and extending the model to incorporate strain-hardening effects for even greater predictive accuracy.

## Figures and Tables

**Table 1:** The compressive indicators at the velocity of 1 m/s.

	Uniform	Gradient-A	Gradient-B
SEA(kJ/kg)	19.22	21.49	19.27
*σ*_m_ (MPa)	30.89	18.50	22.13
*σ*_pl_ (MPa)	23.52	33.97	29.20
*ε*_d_	0.628	0.771	0.742

**Table 2:** The compressive indicators at the velocity of 10 m/s.

	Uniform	Gradient-A	Gradient-B
SEA(kJ/kg)	20.29	21.95	21.64
*σ*_m_ (MPa)	38.65	26.23	33.90
*σ*_pl_ (MPa)	25.07	34.38	31.68
*ε*_d_	0.678	0.758	0.765

**Table 3:** The compressive indicators at the velocity of 100 m/s.

	Uniform	Gradient-A	Gradient-B
SEA(kJ/kg)	34.98	41.22	36.01
*σ*_m_ (MPa)	119.94	74.97	169.49
*σ*_pl_ (MPa)	52.65	67.64	52.50
*ε*_d_	0.802	0.851	0.822

## Data Availability

The original contributions presented in this study are included in the article material. Further inquiries can be directed to the corresponding author.

## Abbreviations

The following abbreviations are used in this manuscript:

NPR	Negative Poisson’s ratio
FE	Finite element
SLM	Selective laser melting
SEA	Specific energy absorption

## Appendix A

By splitting the one-third analytical model of the unit cell in Figure 17 at node-a, forces *P*_1_, *P*_2_, and *F_x_*, and moment *M*_a_ can be generated. For strut-ac + bc, the displacement at *x*-coordinate of node-a can be calculated by applying Castigliano’s second theorem, as follows:

(A1) $$\begin{array}{l} \delta_{a}^{x}=\int_{0}^{l_{2}}\frac{P_{1}x\cos\theta_{2}-F_{x}x\sin\theta_{2}-M_{a}}{EI}\left(-x\sin\theta_{2}\right)dx+\int_{0}^{l_{1}}\frac{P_{1}x\cos\theta_{1}-F_{x}x\sin\theta_{1}-M_{a}}{EI}\left(-x\sin\theta_{1}\right)dx\\ \;=\frac{1}{EI}\left[\left(\frac{l_{2}^{3}}{3}\sin^{2}\theta_{2}+\frac{l_{1}^{3}}{3}\sin^{2}\theta_{1}\right)F_{x}+\left(\frac{l_{2}^{2}}{2}\sin\theta_{2}+\frac{l_{1}^{2}}{2}\sin\theta_{1}\right)M_{a}-\left(\frac{l_{2}^{3}}{6}\sin2\theta_{2}+\frac{l_{1}^{3}}{6}\sin2\theta_{1}\right)P_{1}\right] \end{array}$$

And,

(A2) $$\delta_{a}^{x}=0$$

The rotation angle of node-a can be calculated by

(A3) $$\begin{array}{l} \theta_{a}=\int_{0}^{l_{2}}\frac{P_{1}x\cos\theta_{2}-F_{x}x\sin\theta_{2}-M_{a}}{EI}\left(-1\right)dx+\int_{0}^{l_{1}}\frac{P_{1}x\cos\theta_{1}-F_{x}x\sin\theta_{1}-M_{a}}{EI}\left(-1\right)dx\\ \;=\frac{1}{EI}\left[\left(\frac{l_{2}^{2}}{2}\sin\theta_{2}+\frac{l_{1}^{2}}{2}\sin\theta_{1}\right)F_{x}+\left(l_{2}+l_{1}\right)M_{a}-\left(\frac{l_{2}^{2}}{2}\cos\theta_{2}+\frac{l_{1}^{2}}{2}\cos\theta_{1}\right)P_{1}\right] \end{array}$$

In addition,

(A4) $$\theta_{a}=0$$

By solving Equations (A2) and (A4) simultaneously, the bending moment *M*_a_ and force *F_x_* can be expressed by *P*_1_, as follows:

(A5) $$\begin{array}{l} F_{x}=\frac{4\left({l_{1}}^{3}\sin2\theta_{1}+{l_{2}}^{3}\sin2\theta_{2}\right)\left(l_{1}+l_{2}\right)-6\left({l_{1}}^{2}\sin\theta_{1}+{l_{2}}^{2}\sin\theta_{2}\right)\left({l_{1}}^{2}\cos^{2}\theta_{1}+{l_{2}}^{2}\cos^{2}\theta_{2}\right)}{8\left({l_{1}}^{3}\sin^{2}\theta_{1}+{l_{2}}^{3}\sin^{2}\theta_{2}\right)\left(l_{1}+l_{2}\right)-6{\left({l_{1}}^{2}\sin\theta_{1}+{l_{2}}^{2}\sin\theta_{2}\right)}^{2}}P_{1}\\ \;=C_{F}P_{1} \end{array}$$

(A6) $$\begin{array}{l} M_{a}=\frac{2\left({l_{1}}^{3}\sin2\theta_{1}+{l_{2}}^{3}\sin2\theta_{2}\right)\left({l_{1}}^{2}\sin\theta_{1}+{l_{2}}^{2}\sin\theta_{2}\right)-\left({l_{1}}^{3}\sin^{2}\theta_{1}+{l_{2}}^{3}\sin^{2}\theta_{2}\right)\left({l_{1}}^{2}\cos^{2}\theta_{1}+{l_{2}}^{2}\cos^{2}\theta_{2}\right)}{6{\left({l_{1}}^{2}\sin\theta_{1}+{l_{1}}^{2}\sin\theta_{2}\right)}^{2}-2\left(l_{1}+l_{2}\right)\left({l_{1}}^{3}\sin^{2}\theta_{1}+{l_{2}}^{3}\sin^{2}\theta_{2}\right)}P_{1}\\ \;=C_{M\;a}P_{1} \end{array}$$

Then, we can obtain

(A7) $$M_{c}=P_{1}l_{2}\cos\theta_{2}-M_{a}-F_{x}l_{2}\sin\theta_{2}$$

And it can be rewritten as *M*_c_ = *C_M_*_c_*P*_1_.

For bar-bc, the displacement at the *x*-coordinate of node-c can be calculated by applying Castigliano’s second theorem, as follows:

(A8) $$\begin{array}{l} \delta_{c}^{x}=\int_{0}^{l_{1}}\frac{P_{1}x\cos\theta_{1}-F_{x}x\sin\theta_{1}-M_{c}}{EI}\left(-x\sin\theta_{1}\right)dx\\ \;=\frac{1}{EI}\left(F_{x}\frac{l_{1}^{3}}{3}\sin^{2}\theta_{1}+M_{c}\frac{l_{1}^{2}}{2}\sin\theta_{1}-P_{1}\frac{l_{1}^{3}}{6}\sin2\theta_{1}\right) \end{array}$$

For strut-ac + bc, the displacement at the *z*-coordinate of node-a is

(A9) $$\begin{array}{l} \delta_{a1}^{z}=\int_{0}^{l_{2}}\frac{P_{1}x\cos\theta_{2}-F_{x}x\sin\theta_{2}-M_{a}}{EI}\left(x\cos\theta_{2}\right)dx+\int_{0}^{l_{1}}\frac{P_{1}x\cos\theta_{1}-F_{x}x\sin\theta_{1}-M_{a}}{EI}\left(x\cos\theta_{1}\right)dx\\ \;=\frac{1}{EI}\left[\left(\frac{l_{2}^{3}}{3}\cos^{2}\theta_{2}+\frac{l_{1}^{3}}{3}\cos^{2}\theta_{1}\right)P_{1}-\left(\frac{l_{2}^{2}}{2}\cos\theta_{2}+\frac{l_{1}^{2}}{2}\cos\theta_{1}\right)M_{a}-\left(\frac{l_{2}^{3}}{6}\sin2\theta_{2}+\frac{l_{1}^{3}}{6}\sin2\theta_{1}\right)F_{x}\right] \end{array}$$

For bar-ab, the displacement of node-a is caused by the compression of bar-ab, and can be characterized by

(A10) $$\delta_{a2}^{z}=\frac{P_{2}h}{EA_{b}/3}$$

The displacement of node-a satisfies the deformation coordination condition as

(A11) $$\delta_{a1}^{z}=\delta_{a2}^{z}$$

Equations (A7) and (A8) can be substituted into Equation (A9). In addition, the total force of node-a is

(A12) $$P_{1}+P_{2}=P$$

Therefore, by solving Equations (A.9) and (A.10) simultaneously, the total force *P* can be expressed by *P*_1_ as follows:

(A13) $$P=P_{1}+\frac{A_{b}P_{1}}{18Ih}\left[2{l_{1}}^{3}\cos^{2}\theta_{1}+2{l_{2}}^{3}\cos^{2}\theta_{2}-C_{F}\left({l_{1}}^{3}\sin2\theta_{1}+{l_{2}}^{3}\sin2\theta_{2}\right)-3C_{M}\left({l_{1}}^{2}\cos\theta_{1}+{l_{2}}^{2}\cos\theta_{2}\right)\right]$$

In another form,

(A14) $$P_{1}=C_{1}P$$

and *P*_2_ = (1 − *C*_1_)*P*.

The effective Poisson’s ratio of the unit cell can be expressed as

(A15) $$v_{zx}=v_{zy}=-\frac{\delta_{c}^{x}/l_{1}\cos\theta_{1}}{\delta_{a1}^{z}/l_{2}\sin\theta_{2}}$$

Introducing Equations (A8) and (A9) into Equation (A15), it can be deduced that

(A16) $$v_{zx}=\frac{\left(F_{x}\frac{l_{1}^{2}}{3}\sin^{2}\theta_{1}+M_{c}\frac{l_{1}}{2}\sin\theta_{1}-P_{1}\frac{l_{1}^{2}}{6}\sin2\theta_{1}\right)\sin\theta_{2}}{\left[\left(\frac{l_{2}^{2}}{3}\cos^{2}\theta_{2}+\frac{l_{1}^{3}}{3l_{2}}\cos^{2}\theta_{1}\right)P_{1}-\left(\frac{l_{2}}{2}\cos\theta_{2}+\frac{l_{1}^{2}}{2l_{2}}\cos\theta_{1}\right)M_{a}-\left(\frac{l_{2}^{2}}{6}\sin2\theta_{2}+\frac{l_{1}^{3}}{6l_{2}}\sin2\theta_{1}\right)F_{x}\right]\cos\theta_{1}}$$
